# Prevention of atherothrombotic events in patients with diabetes mellitus: from antithrombotic therapies to new-generation glucose-lowering drugs

**DOI:** 10.1038/s41569-018-0080-2

**Published:** 2018-09-24

**Authors:** Giuseppe Patti, Ilaria Cavallari, Felicita Andreotti, Paolo Calabrò, Plinio Cirillo, Gentian Denas, Mattia Galli, Enrica Golia, Ernesto Maddaloni, Rossella Marcucci, Vito Maurizio Parato, Vittorio Pengo, Domenico Prisco, Elisabetta Ricottini, Giulia Renda, Francesca Santilli, Paola Simeone, Raffaele De Caterina, on behalf of the Working Group on Thrombosis of the Italian Society of Cardiology

**Affiliations:** 1 Department of Life, Health and Environmental Sciences, University of L’Aquila, L’Aquila, Italy; 2 Unit of Cardiovascular Science, Campus Bio-Medico University, Rome, Italy; 3 Cardiovascular and Thoracic Department, Fondazione Policlinico Universitario A. Gemelli IRCCS, Rome, Italy; 4 Department of Cardio-thoracic and Respiratory Sciences, University of Campania “Luigi Vanvitelli”, Naples, Italy; 5 Department of Advanced Biomedical Sciences, University Federico II, Naples, Italy; 6 Department of Cardiac, Thoracic and Vascular Sciences, University of Padova, Padova, Italy; 7 Department of Medicine, Unit of Endocrinology and Diabetes, Campus Bio-Medico University, Rome, Italy; 8 Department of Experimental and Clinical Medicine, University of Florence, Florence, Italy; 9 Cardiology Unit, Madonna del Soccorso Hospital, San Benedetto del Tronto, Italy; 10 Politecnica Delle Marche University, San Benedetto del Tronto, Italy; 11 Institute of Cardiology, G. d’Annunzio University, Chieti, Italy; 12 Department of Medicine and Aging, G. d’Annunzio University, Chieti, Italy; 13 Fondazione G. Monasterio, Pisa, Italy

**Keywords:** Thrombosis, Type 1 diabetes, Type 2 diabetes, Preventive medicine, Drug therapy

## Abstract

Diabetes mellitus is an important risk factor for a first cardiovascular event and for worse outcomes after a cardiovascular event has occurred. This situation might be caused, at least in part, by the prothrombotic status observed in patients with diabetes. Therefore, contemporary antithrombotic strategies, including more potent agents or drug combinations, might provide greater clinical benefit in patients with diabetes than in those without diabetes. In this Consensus Statement, our Working Group explores the mechanisms of platelet and coagulation activity, the current debate on antiplatelet therapy in primary cardiovascular disease prevention, and the benefit of various antithrombotic approaches in secondary prevention of cardiovascular disease in patients with diabetes. While acknowledging that current data are often derived from underpowered, observational studies or subgroup analyses of larger trials, we propose antithrombotic strategies for patients with diabetes in various cardiovascular settings (primary prevention, stable coronary artery disease, acute coronary syndromes, ischaemic stroke and transient ischaemic attack, peripheral artery disease, atrial fibrillation, and venous thromboembolism). Finally, we summarize the improvements in cardiovascular outcomes observed with the latest glucose-lowering drugs, and on the basis of the available evidence, we expand and integrate current guideline recommendations on antithrombotic strategies in patients with diabetes for both primary and secondary prevention of cardiovascular disease.

## Abstract (web summary)

Patients with diabetes mellitus have a prothrombotic status that increases the risk of cardiovascular events and worsens prognosis after these events. In this Consensus Statement, the Working Group on Thrombosis of the Italian Society of Cardiology proposes antithrombotic strategies for patients with diabetes in various cardiovascular settings.

## Introduction

Diabetes mellitus is an important risk factor for a first cardiovascular event and for worse outcomes after a cardiovascular event has occurred. Cardiovascular disease in diabetes is a progressive process characterized by early endothelial dysfunction, oxidative stress, and vascular inflammation leading to monocyte recruitment and formation of foam cells and fatty streaks, which cause development of atherosclerotic plaques over years^1^. Compared with atherosclerotic plaques from individuals without diabetes, those from patients with diabetes are more vulnerable (rupture-prone), and therefore, these plaques are at increased risk of developing superimposed thrombosis because of increased amounts of soft extracellular lipids, inflammation, and prothrombotic milieu; this situation predisposes patients with diabetes to acute cardiovascular events^1^. Consequently, in principle, aggressive antithrombotic therapies might be associated with greater clinical benefit in patients with diabetes than in those without the condition. However, the ischaemic protection provided by antithrombotic drugs must be weighed against the drug-related bleeding risk.

This Consensus Statement from the Working Group on Thrombosis of the Italian Society of Cardiology provides up-to-date recommendations on primary and secondary prevention of atherothrombotic events in patients with diabetes. We explore the mechanisms of platelet and coagulation activity and analyse the current data on the risk–benefit balance of antiplatelet therapy for primary prevention of cardiovascular disease in patients with diabetes. We evaluate the differential protection provided by different antithrombotic therapies in secondary prevention of cardiovascular disease according to diabetic status in various settings (stable coronary artery disease (CAD), acute coronary syndrome (ACS), ischaemic cerebrovascular events, peripheral artery disease (PAD), and venous thromboembolism (VTE)). We also analyse the relationship between diabetes and thromboembolic risk in atrial fibrillation (AF) and the efficacy and safety of the non-vitamin K antagonist oral anticoagulants (NOACs) in patients with diabetes and AF. On the basis of the available evidence, we propose antithrombotic strategies that expand and integrate current guideline recommendations for patients with diabetes^2–4^ (Table 1). Finally, we discuss the cardiovascular outcomes data from randomized, controlled trials on the new classes of glucose-lowering drugs.

**Table 1:** Recommendations on antithrombotic treatment in patients with diabetes mellitus

Indication	ESC/EASD (2013)^4^	AHA/ADA (2015)^3^	ADA (2018)^2^	Recommendations in this Consensus Statement
Primary prevention of CVD	• Low CV risk: aspirin not recommended (class III, LoE A) • High CV risk: aspirin can be considered on an individual basis (class IIb, LoE C)	• 10-year CV risk ≥10%: aspirin reasonable if no increased risk of bleeding (AHA class IIa, LoE B; ADA grade C) • 10-year CV risk 5–10%: aspirin can be considered if no increased risk of bleeding (AHA class IIb, LoE C; ADA grade E) • 10-year CV risk <5%: aspirin not recommended (AHA class III, LoE C; ADA grade C)	Aspirin can be considered in patients with diabetes aged ≥50 years with one additional CV risk factor and no increased risk of bleeding	Patients with 10-year CV risk >10% should initiate aspirin if aged ≥50 years without a high risk of bleeding, and aspirin therapy can be considered if aged 50–70 years with a family history of colorectal cancer
Secondary prevention of CVD in patients with stable CAD	Aspirin 75–160 mg per day	NA	Aspirin 75–162 mg per day^a^	• Aspirin 75–100 mg per day or clopidogrel 75 mg per day • Consider rivaroxaban 2.5 mg twice daily in addition to aspirin
Secondary prevention of CVD in patients with ACS	• DAPT for 1 year^b^ • If PCI: prasugrel or ticagrelor preferred	NA	• DAPT for 1 year^b^ • If PCI: clopidogrel, prasugrel, or ticagrelor • If no PCI: clopidogrel or ticagrelor • DAPT prolongation might have benefits	Consider DAPT prolongation beyond 1 year with aspirin plus ticagrelor 60 mg twice daily in selected patients without high bleeding risk and with high ischaemic risk
Secondary prevention of CVD in patients with ischaemic stroke or transient ischaemic attack	Aspirin 75–160 mg per day	NA	Aspirin 75–162 mg per day^a^	• Aspirin (50–325 mg per day) or clopidogrel (75 mg per day) • DAPT can be considered in selected patients during the first month after a nondisabling stroke
Secondary prevention of CVD in patients with PAD	Antiplatelet therapy recommended in symptomatic PAD	NA	Aspirin 75–162 mg per day^a^	• Aspirin (75–100 mg per day) or clopidogrel (75 mg per day) in symptomatic PAD • Consider rivaroxaban 2.5 mg twice daily in addition to aspirin • DAPT after lower-extremity revascularization can be considered
Prevention of thromboembolic events in AF	• OAC should be used in all patients with AF if not contraindicated • If unable to use OAC, aspirin plus clopidogrel should be considered	NA	NA	• If CHA_2_DS_2_-VASc score = 1, OAC initiation should be tailored on an individual basis • If CHA_2_DS_2_-VASc score ≥2, OAC is recommended, preferring a NOAC
Prevention and treatment of venous thromboembolic events	NA	NA	NA	OAC up to 3–6 months after the event, preferring a NOAC

ACS, acute coronary syndrome; ADA, American Diabetes Association; AF, atrial fibrillation; CAD, coronary artery disease; CV, cardiovascular; CVD, cardiovascular disease; DAPT, dual antiplatelet therapy; EASD, European Association for the Study of Diabetes; LoE, level of evidence; NA, not applicable; NOAC, non-vitamin K antagonist oral anticoagulant; OAC, oral anticoagulant therapy; PAD, peripheral artery disease; PCI, percutaneous coronary intervention.

^a^This recommendation refers to patients with any atherosclerotic cardiovascular disease.

^b^After 1 year, continue lifelong aspirin.

## Platelet function and reactivity

**Fig. 1:**
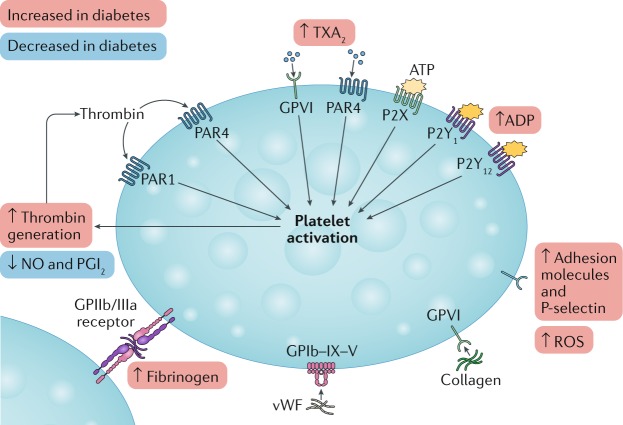
Pathways leading to increased platelet aggregability in diabetes mellitus. Increased platelet reactivity in diabetes involves higher levels of thrombin generation, increased production of thromboxane A_2_ (TXA_2_), hyperresponsiveness of proteinase-activated receptor 4 (PAR4) to thrombin and TXA_2_, and increased platelet membrane expression of P-selectin, adhesion molecules, and glycoprotein (GP) IIb/IIIa. Signalling of P2Y purinoceptor 12 (P2Y_12_ receptor) — the main platelet receptor for ADP — is also increased. Vascular synthesis of nitric oxide (NO) and prostaglandin I_2_ (PGI_2_; also known as prostacyclin) is decreased, and the production of reactive oxygen species (ROS) is increased. P2X, P2X purinoceptor; P2Y_1_, P2Y purinoceptor 1; vWF, von Willebrand factor.

Platelets of individuals with diabetes, compared with those of healthy controls, have dysregulation at both the receptor and the intracellular signal transduction levels, leading to hyperreactive adhesion, activation, degranulation, and aggregation^5^. Reduced insulin sensitivity causes increased signalling of P2Y purinoceptor 12 (P2Y_12_ receptor), the main platelet receptor for ADP^6^ (Box 1; Fig. 1). Hyperglycaemia and associated conditions, such as obesity, dyslipidaemia, and inflammation, modulate this phenotype^5^.

In vitro studies from patients with diabetes have shown increased platelet membrane expression of P-selectin and glycoprotein IIb/IIIa (GPIIb/IIIa), as well as increased platelet response to adrenaline and thrombin^5^. Impairment of the arachidonic acid pathway accounts for platelet dysfunction in vivo, which can be improved through suppression of platelet cyclooxygenase 1 (COX1; also known as prostaglandin G/H synthase 1)^7^.

Platelets from patients with diabetes show increased turnover, resulting in a higher number of reticulated platelets, and increased adhesion to endothelial cells^8^. A reduction in platelet fluidity occurs through changes in membrane lipid structure or glycation of membrane proteins^5^. Furthermore, platelets from patients with diabetes have an increased expression of adhesion molecules, such as CD63 antigen, integrin α2 (CD49B), platelet endothelial cell adhesion molecule 1 (CD31), platelet glycoprotein 4 (CD36), and P-selectin (CD62P), as documented by flow cytometry^9^.

Another important determinant of platelet dysfunction in diabetes is the reduced vascular production of nitric oxide (NO) and prostaglandin I_2_ (PGI_2_; also known as prostacyclin), which physiologically inhibit platelet aggregation^8^. Other reactive oxygen species (ROS) might also contribute to in vivo platelet hyperactivation in diabetes^8^.

In patients with ACS or those undergoing coronary stent implantation, the recurrence of cardiovascular events is not infrequent despite using the current standard of care, which is dual antiplatelet therapy (DAPT) comprising acetylsalicylic acid (commonly referred to as aspirin) and a P2Y_12_-receptor blocker. Platelet-function studies have revealed that treatment with clopidogrel is associated with an overall modest level of P2Y_12_-receptor inhibition with interindividual variability, even when high loading doses are used^10,11^. ADP-induced high platelet reactivity (HPR) when taking clopidogrel has been identified as a marker of vascular risk in an international consensus document^12^.

Patients with diabetes have a more pronounced impairment in their response to clopidogrel than individuals without diabetes^13–17^. This finding was demonstrated in ex vivo testing of platelet reactivity and was also derived by subgroup analyses of clinical trials, in which patients with diabetes who were undergoing percutaneous coronary intervention (PCI) and receiving clopidogrel had a worse clinical course and a higher incidence of stent thrombosis than patients without diabetes^13–16^. The exact mechanism of this phenomenon remains unknown. Reduced insulin sensitivity in diabetes might cause increased signalling of P2Y_12_ receptors; in addition, insulin might physiologically reduce platelet aggregation by inhibiting the P2Y_12_ pathway through platelet insulin receptors, and this mechanism might be impaired in patients with diabetes^18^. Finally, diabetes is known to attenuate cytochrome P450 activity in animal models and in humans^19–21^; accordingly, in patients with diabetes who are receiving clopidogrel, HPR has been shown to be correlated with lower concentrations of the clopidogrel active metabolite^22^.

Another area of research is investigating the possible role of HPR in response to other platelet agonists, in particular arachidonic acid, which predominantly mirrors the effects of aspirin on platelet inhibition. Diabetes has been associated with HPR in response to arachidonic acid; aspirin-naive patients with diabetes seem to have elevated levels of platelet-derived thromboxane, and aspirin might be less effective in inhibiting thromboxane synthesis in patients with diabetes than in individuals without diabetes^23^. In the randomized ASPECT study^24^, in which multiple methods were used to assess responsiveness to aspirin (81 mg daily), patients with diabetes had greater platelet aggregability than those without diabetes, as measured by the VerifyNow assay (Accriva Diagnostics), urinary 11-dehydro-thromboxane B_2_ levels, and collagen-induced light transmittance aggregometry. Of note, increasing the dose of aspirin to >81 mg daily in patients with diabetes reduced platelet function and the prevalence of impaired response to the drug to values similar to those in patients without diabetes; this finding suggests that, in diabetes, low-dose aspirin does not provide adequate platelet inhibition and that higher aspirin dosing reduces the prevalence of low-responder patients^24^. In particular, a systematic review combining data from 31 studies showed that patients with diabetes were 36% more likely to have an impaired response to aspirin than those without diabetes and were 70% more likely to have a low drug response with aspirin 100 mg than with 101–325 mg daily^25^. A possible mechanism underlying HPR when receiving aspirin in patients with diabetes might be a faster recovery of platelet COX1 activity leading to incomplete thromboxane inhibition during the 24-h dosing interval. Indeed, aspirin 100 mg twice daily completely reversed the abnormal thromboxane B_2_ recovery in patients with diabetes^26,27^.

Box 1 Mechanisms of platelet dysfunction in diabetes mellitus **Hyperglycaemia**

- Increased expression of platelet adhesion molecules
- Increased expression of P-selectin, glycoprotein Ib, and glycoprotein IIb/IIIa
- Reduced platelet membrane fluidity owing to changes in the lipid composition of the membrane or glycation of membrane proteins
- Activation of protein kinase C

**Insulin resistance or deficiency**

- Impaired response to nitric oxide and prostacyclin
- Increased formation of reactive oxygen species
- Increased intracellular Ca^2+^ and degranulation

**Other cellular abnormalities**

- Upregulation of platelet P2Y purinoceptor 12 (P2Y_12_ receptor) signalling, which decreases cAMP levels and lowers insulin responsiveness
- Increased generation of thrombin
- Increased production of thromboxane A_2_ from arachidonic acid metabolism
- Accelerated platelet turnover, resulting in increased numbers of reticulated platelets

**Associated metabolic conditions**

- Obesity
- Dyslipidaemia
- Inflammation

## Coagulation activity

**Fig. 2:**
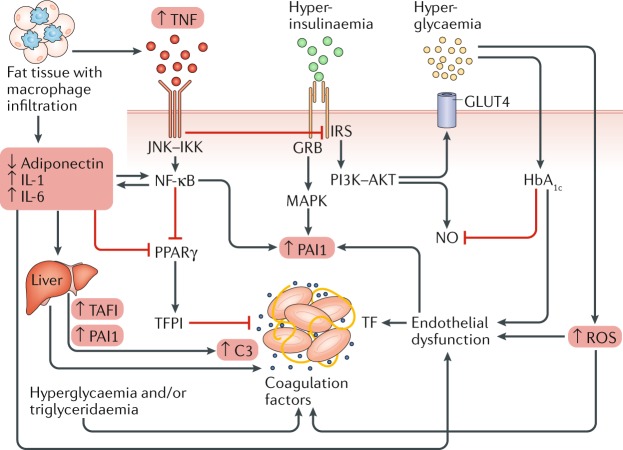
Intracellular pathways underlying procoagulant patterns in diabetes mellitus. Fat tissue produces less adiponectin and is infiltrated by macrophages that release tumour necrosis factor (TNF), IL-1, and IL-6. This inflammatory state increases the synthesis of plasminogen activator inhibitor 1 (PAI1) and tissue factor (TF) by endothelial cells, as well as coagulation factors, carboxypeptidase B2 (also known as thrombin-activable fibrinolysis inhibitor; TAFI), PAI1, and acute phase proteins, such as complement C3, by the liver. TNF blocks the vasculoprotective insulin pathway involving insulin receptor substrate (IRS)–phosphoinositide 3-kinase (PI3K)–RACα serine/threonine-protein kinase (AKT) and activates inflammation through the signalling pathway involving c-Jun N-terminal kinase (JNK)–inhibitor of nuclear factor-κB kinase (IKK)–nuclear factor-κB (NF-κB). Impaired IRS–PI3K–AKT transduction alters nitric oxide (NO) and insulin-responsive glucose transporter type 4 (GLUT4; also known as SLC2A4) function, whereas the prothrombotic insulin pathway involving growth factor receptor-bound protein (GRB)–mitogen-activated protein kinase (MAPK) remains effective. Inflammation also blunts peroxisome proliferator-activated receptor-γ (PPARγ)-mediated synthesis of the anticoagulant tissue factor pathway inhibitor (TFPI). Hyperglycaemia increases production of reactive oxygen species (ROS) and contributes to endothelial dysfunction. Increased levels of glycated haemoglobin (HbA_1c_) alter the physiological transport and release of NO. Hyperglycaemia and triglyceridaemia favour the synthesis of coagulation factors and PAI1.

Multiple pathophysiological mechanisms might contribute to the prothrombotic environment associated with type 2 diabetes (Fig. 2; Table 2), a condition characterized by hyperglycaemia, hyperinsulinaemia, low-grade inflammation, and raised plasma triglyceride levels.

**Table 2:** Thrombotic and fibrinolytic factors in diabetes mellitus

Factor	Function	Change in levels with diabetes	Effect
Tissue factor–coagulation factor VII	Initiates clot formation	↑	↑ Thrombosis
Fibrinogen	Forms fibrin clot	↑ (and ↑ glycation)	↑ Thrombosis and ↑ clot density
Thrombin	Converts fibrinogen to fibrin	↑	↑ Thrombosis and ↑ clot stability
Plasminogen activator inhibitor 1	Inhibits production of plasmin	↑	↓ Fibrinolysis
Plasminogen or plasmin	Breaks down fibrin clot	↓ (and ↑ glycation)	↓ Fibrinolysis and ↑ clot density
Carboxypeptidase B2	Inhibits fibrin breakdown	↑	Delayed clot lysis
Tissue-type plasminogen activator	Converts plasminogen to plasmin	↓	↓ Fibrinolysis
Complement C3	Complement system	↑	↑ Clot density
Glycated haemoglobin A_1c_	Reflects hyperglycaemic milieu	↑	↓ Nitric oxide bioavailability
Peroxisome proliferator-activated receptor-γ	Nuclear transcription factor	↓	↓ Inhibitor of the tissue factor pathway

Hyperglycaemia has direct effects on gene transcription of coagulation factors, and hyperglycaemia-induced oxidative stress alters the natural vasculoprotective endothelial glycocalyx^28,29^. Insulin and proinsulin-like molecules in experimental systems promote the expression and secretion of plasminogen activator inhibitor 1 (PAI1) by hepatocytes and endothelial cells^29,30^.

Quantitative changes in coagulation factors in patients with diabetes involve higher plasma levels of fibrinogen (the soluble precursor of solid fibrin), coagulation factor VII, and coagulation factor XII; increased endothelial expression of tissue factor and tissue factor–coagulation factor VIIa complex activity; and a reduction in the anticoagulant protein tissue factor pathway inhibitor (TFPI)^28,29^. These changes culminate in increased thrombin generation and fibrin formation. Quantitative changes of fibrinolytic factors include increased plasma levels of PAI1 and carboxypeptidase B2 (also known as thrombin-activable fibrinolysis inhibitor)^29,30^. Levels of tissue-type plasminogen activator (t-PA) are also altered, with raised antigen concentrations and reduced t-PA activity^29,30^.

Qualitative changes of haemostatic factors include the glycation and oxidation of fibrinogen and plasminogen, as well as the incorporation of antiplasmin, PAI1, carboxypeptidase B2, and complement C3 into the fibrin mesh, resulting in a denser clot structure and in delayed spontaneous clot lysis^28,29^. Increased thrombin generation activates coagulation factor XIII, which cross links and further stabilizes the fibrin network^29^. Moreover, glycation of haemoglobin in patients with diabetes alters the physiological transport and release of NO by haemoglobin itself, with impaired peripheral vasodilatation, increased insulin resistance, and pro-inflammatory and prothrombotic effects^31^. An overall hypercoagulable state is demonstrated by the shortening of activated partial thromboplastin time in patients with diabetes compared with individuals without diabetes^29^.

In summary, our current understanding of the pathophysiological steps implicated in the increased thrombotic risk of patients with diabetes includes increased thrombin and fibrin generation, delayed clot lysis, and reduced NO bioavailability. Future therapies targeting the coagulation, fibrinolysis, and NO systems, rather than platelets alone, might open new frontiers in the prevention and treatment of cardiovascular diseases in these patients.

## Primary prevention of cardiovascular disease

Data from Haffner and colleagues supported the concept that patients with diabetes but without previous CAD have a risk of future CAD events similar to that of patients without diabetes but with previous symptomatic CAD^32^; therefore, aspirin was considered to be of value for primary prevention in patients with diabetes. However, a meta-analysis of 13 studies, involving a total of 45,108 patients, has subsequently shown that the cardiovascular risk of patients with diabetes without previous cardiovascular events is significantly lower than that of patients without diabetes who have previous cardiovascular disease, refuting the notion that diabetes is an equivalent risk factor to CAD^33^. Nevertheless, the risk of cardiovascular events in patients with diabetes is twofold to fourfold greater than in individuals of the same age and sex without diabetes^34,35^; this risk should be balanced against the bleeding risk associated with aspirin. An individual patient-level meta-analysis by the Antithrombotic Trialists’ (ATT) Collaboration indicated a similar effect of aspirin on primary prevention of major cardiovascular events in individuals with or without diabetes, with a risk ratio (RR) of 0.88 (95% CI 0.67–1.15) and 0.87 (95% CI 0.79–0.96), respectively^36^.

Other studies have involved individuals with higher cardiovascular risk profiles than those included in the ATT meta-analysis. In the JPPP trial^37^, individuals aged 60–85 years with hypertension, dyslipidaemia, or diabetes were randomly assigned to aspirin 100 mg daily or placebo. The 5-year primary outcome event rate (cardiovascular death, nonfatal stroke, and nonfatal myocardial infarction (MI)) was not different in the two groups, but treatment with aspirin significantly increased the risk of extracranial bleeding. Conversely, in the CLIPS trial^38^, which was performed in patients with peripheral atherosclerosis but without a history of CAD, low-dose aspirin prevented serious vascular events compared with placebo, including in the subgroups with type 2 diabetes.

Six randomized clinical studies, in which patients with diabetes were considered as subgroups (1–22% of the overall trial cohort), have not provided definitive results on the benefit of aspirin in primary prevention of cardiovascular disease^39–44^. Additionally, three randomized trials on primary prevention of cardiovascular disease were conducted specifically in patients with diabetes^45–47^, one of them with a 10-year follow-up^48^. The oldest study used higher aspirin doses (650 mg daily) versus placebo^45^, whereas subsequent studies used lower aspirin doses (100 mg, or 81–100 mg daily in the JPAD study)^46,47^; none of the studies showed a significant reduction in cardiovascular events with aspirin use. A subsequent analysis of these nine studies found that aspirin treatment in patients with diabetes was associated with nonsignificant reductions in CAD (−9%) and cerebrovascular events (−11%) compared with control^35^. Other meta-analyses produced similar estimates^49,50^. Overall, these results suggest that aspirin, as currently prescribed, produces only a modest reduction in the risk of cardiovascular events when used for primary prevention in patients with diabetes^51^.

A word of caution concerns the heterogeneity and limitations of the primary prevention studies, including underpowering for sample size, differences in study designs, primary end points, risk profiles, and aspirin regimens, high rates of aspirin discontinuation during follow-up, and non-uniform reports of statin treatment prevalence. Despite these biases, the less-than-expected clinical benefit of aspirin in patients with diabetes might be explained by the presence of multiple thrombotic pathways involved in the pathogenesis of cardiovascular events and by the aforementioned platelet features (including residual hyperreactivity or increased turnover, especially in individuals with poor glycaemic control)^52^. Various aspirin formulations have been tested in attempts to improve its efficacy and/or limit adverse effects. An enteric-coated formulation was associated with increased rates of low responsiveness to the drug owing to reduced bioavailability compared with uncoated aspirin; this phenomenon might contribute to aspirin pseudo-resistance, with possibly lower clinical benefits in patients with diabetes receiving the coated formulation^53^. An extended-release, 162.5 mg formulation of aspirin was shown to provide a sustained antiplatelet effect over 24 h in patients with type 2 diabetes and multiple cardiovascular risk factors, with a favourable tolerability profile^54^; however, no extensive clinical data are available with this formulation. The previously mentioned increase in aspirin dose has been suggested to improve the effectiveness of the drug, but with inconsistent results and uncertain net benefit, the latter largely owing to the increased risk of adverse effects as a consequence of the reduced production of gastric prostaglandins, which protect against gastrointestinal bleeding, compared with the use of low doses of aspirin^24,55^. As discussed above, a twice-daily administration of low-dose aspirin has been tested in pharmacodynamic studies in patients with diabetes and concomitant CAD because this regimen might provide greater platelet inhibition than once-daily administration^26,56^; however, evidence of improved cardiovascular outcomes with twice-daily administration is still needed and is currently under investigation in the randomized ANDAMAN trial^57^.

Regarding the bleeding risk of aspirin, a population-based study found a significant excess of both gastrointestinal and intracranial bleeding with aspirin compared with no aspirin use in individuals without diabetes but not in patients with diabetes^58^; this finding indirectly suggests a lower suppression of platelet function by standard aspirin doses in those with diabetes than in those without diabetes. Taken together, the overall available data on aspirin in patients with diabetes indicate a modest relative reduction in the risk of cardiovascular events in primary prevention offset against a 55% relative increase in the risk of extracranial bleeding, which is mainly gastrointestinal^58,59^. Therefore, we recommend that patients aged ≥50 years with a 10-year cardiovascular risk >10% according to the Systematic Coronary Risk Estimation (SCORE) risk charts^59^ and without high bleeding risk should initiate aspirin. Moreover, long-term follow-up of studies of aspirin in primary prevention of cardiovascular disease showed that low doses of this drug are associated with a reduction in colorectal cancer incidence and mortality from 5 years onwards^60^. Although data on cancer prevention with the use of aspirin specifically in patients with diabetes are lacking, this issue needs to be addressed, because patients with type 2 diabetes have an increased risk of some types of cancer, particularly of the pancreas and the colon–rectum^61^. Table 3 summarizes our recommendations on the use of aspirin in adults with diabetes and no pre-existing cardiovascular disease. Two primary prevention trials in diabetes (ASCEND^62^ and ACCEPT-D^63^) will help to assess the benefit–risk profile of low-dose aspirin in preventing multiple outcomes. The ASCEND trial^62^, in which adults with diabetes but no evident cardiovascular disease were randomly assigned to receive 100 mg of aspirin daily or placebo, was published in August 2018; in this study, the absolute benefits of aspirin in preventing serious vascular events were largely counterbalanced by the increased risk of bleeding.

**Table 3:** Recommendations on antithrombosis for prevention of cardiovascular disease in diabetes mellitus

Indication	Subgroup	Working Group recommendations
*Primary prevention of cardiovascular disease*		
10-year cardiovascular risk	<5%	• Antiplatelet therapy is not recommended
	5–10%	• Any decision on aspirin initiation should take into account the individual risk of bleeding, and patients at high risk of bleeding should not be treated
	>10%	• Patients aged ≥50 years without a high risk of bleeding should initiate aspirin (75–100 mg daily) • Aspirin therapy (75–100 mg daily) can be considered in patients aged 50–70 years with a family history of colorectal cancer
*Secondary prevention of cardiovascular disease*		
Coronary artery disease	Stable	• Lifelong aspirin (75–100 mg daily) or clopidogrel (75 mg daily) is recommended as first-line, single antiplatelet therapy • DAPT (aspirin plus clopidogrel) is indicated after stent implantation^a^: DAPT is generally indicated for 6 months after stent implantation, regardless of the stent type (drug-eluting or bare-metal stent); a shorter duration is considered in selected patients at low coronary risk and high bleeding risk, whereas a longer duration is considered in selected patients at low bleeding risk and high coronary risk • DAPT is indicated for ≥12 months after bioresorbable vascular scaffold implantation^a^ • DAPT should be avoided in medically managed patients • Consider the addition of rivaroxaban 2.5 mg twice daily to aspirin therapy
	Acute coronary syndrome	• Lifelong aspirin (150–300 mg loading dose and then 75–100 mg daily) is recommended • DAPT is indicated after acute coronary syndrome^a^: addition of a P2Y_12_ inhibitor (prasugrel or ticagrelor as first choice^b^) to aspirin is recommended in all patients for 1 year after the event • DAPT prolongation beyond 1 year (aspirin plus ticagrelor 60 mg twice daily^a^) is indicated in selected patients without high bleeding risk and with high ischaemic risk^c^
Ischaemic stroke	All	• Aspirin (50–325 mg daily) or clopidogrel (75 mg daily) is recommended • DAPT (aspirin plus clopidogrel) can be considered only in selected patients during the first month after a nondisabling stroke, especially in patients at low bleeding risk • Aspirin plus ERDP, although effective, is not indicated owing to high incidence of ERDP-related adverse effects
Peripheral artery disease	Symptomatic	• Lifelong aspirin (75–100 mg daily) or clopidogrel (75 mg daily) alone is recommended • Routine DAPT is not indicated • Consider rivaroxaban 2.5 mg twice daily in addition to aspirin • DAPT after lower-extremity revascularization can be considered to prevent limb-related events
	Asymptomatic with ABI <0.90	• Aspirin or clopidogrel alone is a reasonable option
	Asymptomatic with ABI ≥0.91	• Routine antiplatelet therapy is not indicated
Atrial fibrillation	CHA_2_DS_2_-VASc score ≥2	• Oral anticoagulant therapy, and preferably a non-vitamin K antagonist oral anticoagulant, is recommended • Aspirin is not indicated
	CHA_2_DS_2_-VASc score = 1	• The choice of whether or not to initiate oral anticoagulant therapy should be tailored on an individual basis, considering the type of diabetes, the duration of diabetes, the burden of atrial fibrillation, and the concomitance of renal failure or left atrial dilatation with low-flow velocities in the left atrial appendage • Aspirin is not indicated

ABI, ankle–brachial index; DAPT, dual antiplatelet therapy; ERDP, extended-release dipyridamole; P2Y_12_, P2Y purinoceptor 12.

^a^Recommendation in agreement with the ESC^177^ and ACC/AHA^178^ guidelines on DAPT, in which no differences in DAPT type or duration are recommended in patients with or without diabetes who have an acute coronary syndrome or are undergoing percutaneous coronary intervention (PCI).

^b^Prasugrel (60 mg loading dose and then 10 mg daily) should be used in patients who are undergoing PCI without an indication for chronic oral anticoagulation, without a history of transient ischaemic attack or stroke, and without a high bleeding risk or severe renal failure; ticagrelor (180 mg loading dose and then 90 mg twice daily) should be used in patients who are either conservatively treated or PCI-treated without an indication for chronic oral anticoagulation and without a history of intracranial bleeding, high bleeding risk, severe renal failure, or concomitant use of strong cytochrome P450 3A4 inhibitors; and clopidogrel (600 mg loading dose, then 75 mg daily) should be used in patients who are either conservatively treated or PCI-treated without active bleeding or when prasugrel or ticagrelor are not available or are contraindicated.

^c^High burden of atherosclerosis, recurrent cardiovascular events, or complex PCI.

## Secondary prevention in CAD

### Stable CAD

The presence of diabetes impairs clinical outcomes in patients with cardiovascular disease, in whom meta-analyses of randomized trials indicate an overall 40% increase in major adverse cardiovascular events (MACE; that is, MI, stroke, or cardiovascular death) compared with patients without diabetes^36,64^. In the setting of secondary prevention of cardiovascular disease in patients with diabetes and stable CAD, data from the ATT Collaboration on approximately 4,500 patients showed a 4.2% absolute reduction in cardiovascular events with aspirin compared with placebo at 60-month follow-up, which was similar to that observed in those without diabetes^36^. Clopidogrel has been extensively investigated in patients with stable CAD undergoing PCI or treated conservatively. Table 4 summarizes the main results of randomized trials evaluating antiplatelet therapy in patients with stable CAD for primary efficacy end points in the overall population and in the subgroup with diabetes. In the CAPRIE trial^65^, patients with established cardiovascular disease were randomly assigned to clopidogrel (75 mg daily) or aspirin (325 mg daily). The subgroup analysis including 3,866 patients with diabetes demonstrated that the use of clopidogrel instead of aspirin prevented 21 adverse events (vascular death, MI, stroke, and hospitalization for ischaemic or bleeding complications) per 1,000 patients per year (compared with 9 events prevented per 1,000 patients per year in individuals without diabetes)^66^. Of note, the cardiovascular protection provided by clopidogrel was even higher in the subgroup of patients with diabetes and receiving insulin therapy (38 events prevented per 1,000 patients per year)^66^.

**Table 4:** Trials of antiplatelet therapy in patients with diabetes mellitus and stable CAD or ACS

Trial	Setting	Therapy	Primary end point	All patients	Events in all patients (%)	Patients with diabetes	Events in patients with diabetes (%)	Refs
CAPRIE	Patients at risk of ischaemic events	Aspirin versus clopidogrel	Vascular death, MI, stroke, or hospitalization for ischaemic or bleeding complications at 36 months	19,185	5.8 versus 5.3 (RRR 8.7%, 95% CI 0.3–16.5%)	3,866	17.7 versus 15.6 (RRR 21%, 95% CI NA)	^65^
CHARISMA	Stable CAD with high atherothrombotic risk	Aspirin plus clopidogrel versus aspirin	Cardiovascular death, MI, or stroke at 28 months	15,603	6.8 versus 7.3 (RR 0.93, 95% CI 0.83–1.05)	6,555	6.7 versus 7.7 without nephropathy; 11.4 versus 12.0 with nephropathy	^67^
CREDO	Elective PCI	Aspirin plus clopidogrel versus aspirin	Death, MI, or stroke at 1 year	2,116	8.3 versus 11.5 (RRR 27.0%, 95% CI 3.9–44.4%)	560	NA	^69^
CURE	Unstable angina or NSTEMI	Aspirin plus clopidogrel versus aspirin	Cardiovascular death, MI, or stroke at 1 year	12,562	9.3 versus 11.4 (RR 0.80, 95% CI 0.72–0.90)	2,840	14.2 versus 16.7 (RR 0.84, 95% CI 0.70–1.02)	^76^
TRITON-TIMI 38	ACS with scheduled PCI	Aspirin plus prasugrel versus aspirin plus clopidogrel	Cardiovascular death, MI, or stroke at 1 year	13,608	9.9 versus 12.1 (HR 0.81, 95% CI 0.73–0.90)	3,146	12.2 versus 17.0 (HR 0.70, 95% CI 0.58–0.85)	^84^
PLATO	ACS	Aspirin plus ticagrelor versus aspirin plus clopidogrel	Cardiovascular death, MI, or stroke at 1 year	18,624	9.8 versus 11.7 (HR 0.84, 95% CI 0.77–0.92)	4,662	14.1 versus 16.9 (HR 0.88, 95% CI 0.76–1.03)	^87^
TRILOGY-ACS	Medically managed patients with ACS	Aspirin plus prasugrel versus aspirin plus clopidogrel	Cardiovascular death, MI, or stroke at 30 months	9,326	13.9 versus 16.0 (HR 0.91, 95% CI 0.79–1.05)	3,539	24.0 versus 25.6 (HR 0.95, 95% CI 0.81–1.11)	^85^
DAPT	Stable CAD or ACS treated with DES implantation	Aspirin plus clopidogrel or prasugrel versus aspirin	Stent thrombosis, death, MI, or stroke at 30 months	9,961	4.3 versus 5.9 (HR 0.71, 95% CI 0.59–0.85)	3,391	6.6 versus 7.0 (HR 0.92, 95% CI 0.71–1.20)	^91^
PEGASUS-TIMI 54	History of MI (past 1–3 years)	Aspirin plus ticagrelor versus aspirin	Cardiovascular death, MI, or stroke at 36 months	21,162	7.8 versus 9.0 (HR 0.84, 95% CI 0.74–0.95)	6,806	10.0 versus 11.6 (HR 0.83, 95% CI 0.69–1.00)	^93^

ACS, acute coronary syndrome; CAD, coronary artery disease; DES, drug-eluting stent; MI, myocardial infarction; NA, not available; NSTEMI, non-ST-segment elevation myocardial infarction; PCI, percutaneous coronary intervention; RR, risk ratio; RRR, relative risk reduction.

The addition of clopidogrel to aspirin in stable patients was evaluated in the secondary prevention cohort (that is, patients with previous MI, stroke, or PAD) of the CHARISMA trial^67^. At 28-month follow-up in the subgroup of patients with diabetes, protection from cardiovascular events with the use of DAPT was no greater in patients with diabetes than in those without diabetes, but the bleeding risk was higher in those with diabetes^68^. Of note, the use of aspirin plus clopidogrel compared with aspirin alone led to a significant reduction in the combined end point including cardiovascular death, MI, or stroke only in patients without diabetic nephropathy (6.7% versus 7.7%; *P* = 0.048), whereas no significant benefit was observed in those with diabetic nephropathy (11.4% versus 12.0%; *P* = 0.84)^68^. Therefore, the current evidence indicates that the net clinical benefit of routinely adding clopidogrel to aspirin in patients with diabetes and stable CAD treated medically is questionable because the modest ischaemic protection is outweighed by the increased risk of bleeding.

The CREDO trial^69^ investigated the effects of preprocedural clopidogrel loading in addition to aspirin in patients undergoing elective PCI. Interestingly, in both the overall population and the subgroup with diabetes, pretreatment with 300 mg clopidogrel led to significant clinical benefit compared with downstream administration of clopidogrel, but only when the loading dose was given ≥6 h before the intervention. This finding essentially reflects the fact that several hours are needed to achieve maximal platelet inhibition after a 300 mg clopidogrel load^70,71^. Interestingly, a meta-analysis showed no reduction in MACE incidence with 12-month versus 6-month DAPT among patients with diabetes undergoing PCI with drug-eluting stent implantation for a variety of coronary syndromes^72^. On the basis of the available data, the antithrombotic strategies in patients with diabetes and different features of stable CAD are shown in Table 3.

The COMPASS trial^73^ investigators explored the safety and efficacy of inhibition of thrombin generation, as provided by rivaroxaban (2.5 mg twice daily) on a background of aspirin therapy or by rivaroxaban (5.0 mg twice daily) alone in patients with stable coronary or peripheral atherosclerotic disease (91% with CAD and 62% with previous MI). This study was prematurely stopped for superiority of the rivaroxaban plus aspirin versus aspirin alone group after a mean follow-up of 23 months in terms of MACE (cardiovascular death, MI, or stroke), all-cause death, and cardiovascular death. Consistent with the overall study results, in patients with diabetes (*n* = 6,922), the addition of rivaroxaban to aspirin resulted in a significantly lower incidence of MACE (HR 0.74, 95% CI 0.61–0.90) with higher rates of major bleeding (HR 1.70, 95% CI 1.25–2.31); the net clinical benefit was significantly in favour of combination therapy, without increased rates of intracranial bleeding. Future guidelines on clinical practice in patients with stable CAD are likely to include recommendations derived from the COMPASS study.

### ACS

The clinical benefit provided by antiplatelet therapy in patients with diabetes and an ACS has been clearly demonstrated. Table 4 reports the main results of randomized trials evaluating antiplatelet therapy in patients with ACS for primary efficacy end points in the overall population and in the subgroup with diabetes. Meta-analysis data have shown that the reduction of MACE with aspirin versus placebo after MI is similar in patients with or without diabetes (from 22% to 18% in those with diabetes and from 16% to 13% in those without diabetes)^36,64^. These data also indicate that, despite aspirin therapy, the recurrence of ischaemic events in patients with diabetes and an ACS is approximately 20% over a mean of 2.5 years^36^. Although a persistently higher platelet reactivity when taking aspirin might partly explain this elevated recurrence rate^74^, the randomized CURRENT-OASIS 7 trial^75^ demonstrated no outcome improvement with higher-dose aspirin (300–325 mg daily versus 75–100 mg daily for 30 days) in a mixed population of patients with or without diabetes who were admitted to hospital for ACS, with no significant interaction between primary outcome and diabetic status.

Additional inhibition of the P2Y_12_ platelet receptor on a background of aspirin therapy, aimed at further reducing adverse events in patients with ACS, was first evaluated in the CURE trial^76^, in which patients with non-ST-segment elevation (NSTE)-ACS already receiving aspirin were randomly assigned to clopidogrel (300 mg loading dose followed by 75 mg daily maintenance dose) or placebo for a mean duration of 9 months. A lower rate of MACE was observed with the combination of clopidogrel and aspirin regardless of diabetic status; although no significant interaction was present, the relative reduction of adverse cardiovascular events at 1 year with clopidogrel in the 2,840 patients with diabetes was numerically lower than the reduction in those without diabetes (14% versus 20% relative risk reduction). This finding might be a result of the more pronounced interindividual variability of clopidogrel responsiveness in diabetes, resulting in a higher prevalence of HPR^77–79^.

As discussed above, the mechanisms behind poor clopidogrel-induced P2Y_12_ inhibition in patients with diabetes might include impaired drug metabolism leading to reduced active metabolite generation as well as dysregulation of the P2Y_12_ signalling pathway^80,81^. The OPTIMUS-3 study^82^ found that, in patients with diabetes, prasugrel was associated with faster and greater platelet inhibition, as well as lesser interindividual variability, than clopidogrel, even when the latter was given at high doses (600 mg loading plus 150 mg daily). Similar pharmacodynamic results were obtained with ticagrelor^83^. Given the pharmacodynamic drawbacks of clopidogrel in patients with diabetes, which are potentially overcome with the newer, more potent P2Y_12_ inhibitors, the subgroup analyses of diabetic populations from phase III studies comparing prasugrel and ticagrelor versus clopidogrel in the setting of ACS are of particular interest.

In the TRITON-TIMI 38 trial^84^, the use of prasugrel instead of standard-dose clopidogrel after PCI for ACS led to a higher relative risk reduction of MACE (cardiovascular death, stroke, and MI) at 15 months in patients with diabetes than in those without diabetes (30% versus 14%; *P* = 0.009 for interaction). Importantly, prasugrel did not increase the rates of major bleeding in the diabetes group. In this trial, the greatest reduction in adverse events with prasugrel was observed in patients receiving insulin therapy (37% relative risk reduction versus 26% in patients with diabetes who were not receiving insulin)^84^. The TRILOGY-ACS study^85^ included patients with NSTE-ACS managed without revascularization and randomly assigned to receive prasugrel or clopidogrel for up to 30 months in addition to aspirin. A subgroup analysis according to diabetes status showed no difference in the incidence of MACE (cardiovascular death, MI, or stroke) with either antiplatelet strategy in patients either with (*n* = 3,539) or without (*n* = 5,767) diabetes^86^. In the PLATO trial^87^, patients with ACS were randomly assigned to receive ticagrelor (180 mg loading dose and then 90 mg twice daily) or clopidogrel (300 or 600 mg loading dose and then 75 mg daily), irrespective of the subsequent therapeutic strategy. A lower rate of MACE at 1 year with ticagrelor was observed both in patients with diabetes and in those without diabetes (12% and 17% relative reductions, respectively). This ischaemic protection largely outweighed the elevation in non-CABG surgery-related major bleeding observed in the ticagrelor group. Of note, the use of ticagrelor had a strong effect in decreasing MACE, all-cause death, and stent thrombosis in patients with diabetes and haemoglobin A_1c_ (HbA_1c_) ≥6%.

A pharmacodynamic comparison of prasugrel versus ticagrelor in patients with diabetes and CAD showed that ticagrelor exerts similar or greater inhibition of ADP-induced platelet reactivity than prasugrel in both the acute and the chronic phases of treatment^88^. A clinical comparison between prasugrel and ticagrelor is currently being investigated in the ongoing ISAR-REACT 5 trial^89^, in which diabetes is a predefined subgroup^90^. On the basis of the available evidence, we recommend consistent and highly effective platelet inhibition with the newer, more potent P2Y_12_ inhibitors for patients with ACS and concomitant diabetes, given their high baseline risk profile; therefore, DAPT with aspirin plus prasugrel or ticagrelor should be the first-line antiplatelet strategy up to 1 year in this setting, especially in patients without high bleeding risk (Table 3).

Given the poorer long-term cardiovascular outcomes after MI in patients with diabetes than in those without diabetes, DAPT prolongation is a particularly relevant issue in this setting. The DAPT study^91^ explored the efficacy and safety of prolonging DAPT beyond 1 year with aspirin plus a thienopyridine (prasugrel or clopidogrel) versus aspirin alone for 18 more months in patients with stable CAD or ACS who had undergone implantation of a drug-eluting stent. In the subgroup analysis according to diabetes status (*n* = 3,391 with and *n* = 8,257 without diabetes), a significant interaction existed between MI risk reduction and prolonged DAPT in favour of patients without diabetes (58% relative reduction versus 28% in patients with diabetes; *P* = 0.02 for interaction)^92^. Regardless of diabetes status, the rate of stent thrombosis was lower and the rate of bleeding events was higher in the DAPT group (stent thrombosis: 75% relative reduction in patients without diabetes versus 53% in patients with diabetes, *P* = 0.21 for interaction; moderate or severe bleeding: 71% relative increase in patients without diabetes versus 47% in patients with diabetes, *P* = 0.61 for interaction)^92^. Of note, withdrawal of thienopyridine in both groups resulted in a consistent numerical increase in early ischaemic events in patients with or without diabetes^92^.

The PEGASUS-TIMI 54 trial^93^ explored the efficacy and safety of DAPT prolongation (with ticagrelor 90 mg or 60 mg twice daily plus aspirin versus aspirin alone) in patients with a history of MI (in the past 1–3 years). The reduction in MACE with either ticagrelor dose was consistent in patients with or without diabetes. Given their higher risk of events, patients with diabetes had a greater absolute benefit from ticagrelor, with a 3-year number needed to treat for MACE (pooling results of both ticagrelor doses) of 67 compared with 91 in those without diabetes. Additionally, in patients with diabetes, ticagrelor reduced the rate of cardiovascular death by 22%. As in patients without diabetes, ticagrelor significantly increased the rates of nonfatal major bleeding in patients with diabetes (HR 2.56, *P* = 0.0004). Regardless of diabetic status, the net clinical outcome was better with the 60 mg than with the 90 mg ticagrelor dose — that is, similar ischaemic protection but lower bleeding risk.

The use of ticagrelor for secondary prevention of cardiovascular disease is being investigated specifically in patients with diabetes in the THEMIS trial^94^, where approximately 20,000 patients with type 2 diabetes and CAD, but without previous MI, are being randomly assigned to either ticagrelor (90 mg or 60 mg twice daily) or placebo for up to 4 years. As we wait for more evidence on the net clinical benefit of more aggressive antithrombotic therapies in patients with diabetes and ACS, especially in the long term, we summarize our therapeutic recommendations, which are based on currently available data, in Table 3.

### Other antiplatelet strategies in CAD

Given the hyperreactivity of platelets in patients with diabetes, other antiplatelet strategies have been tested to improve cardiovascular outcomes in these individuals with CAD. Specifically, additional drugs have been investigated as adjunctive treatment to aspirin alone or to aspirin plus a P2Y_12_ inhibitor (triple therapy) to achieve a more complete, multi-pathway platelet inhibition.

Cilostazol, a selective and reversible inhibitor of phosphodiesterase 3 that has effects on various cell lines, including platelets, by increasing intracellular cAMP concentrations^95^, was evaluated in pharmacodynamic studies as part of a triple-therapy strategy in patients with diabetes and CAD. The OPTIMUS-2 study^96^ found that the addition of cilostazol to DAPT improved inhibition of platelet P2Y_12_ signalling. Moreover, cilostazol might decrease platelet aggregability in patients with diabetes and HPR when taking clopidogrel^97^. Clinical benefits of cilostazol in addition to DAPT were observed in a nonrandomized study of Asian patients undergoing PCI, mainly in terms of reduced rates of target-lesion revascularization and stent thrombosis^98^; in randomized studies, this improvement in outcomes was found to be greatest for patients with diabetes^99–101^. Other investigations have evaluated the effects of cilostazol in the setting of PCI for ACS. In the retrospective Korean Acute MI Registry, 4,203 patients with ST-segment elevation MI undergoing primary PCI received either DAPT (aspirin plus clopidogrel) or triple therapy (DAPT plus cilostazol)^102^; the latter strategy was associated with a significantly lower incidence of cardiac death, all-cause death, and MACE at 8-month follow-up. Interestingly, subgroup analysis showed that patients who were older (aged >65 years), female, or had diabetes derived greater benefit from triple therapy^102^. In another study, 1,212 Chinese patients with ACS undergoing PCI were randomly assigned to DAPT (aspirin plus clopidogrel) or DAPT plus cilostazol for 6 months^103^. Triple therapy resulted in a 35% relative reduction in MACE, without excess bleeding; the subgroup of 263 patients with diabetes derived particular benefit from the addition of cilostazol (HR 0.47, 95% CI 0.23–0.96)^103^.

Other attractive antiplatelet agents are thromboxane antagonists that potentially block the interaction of both aspirin-sensitive and aspirin-insensitive agonists, theoretically providing potent platelet inhibition, especially in patients with diabetes who show both platelet hyperreactivity and increased thromboxane-dependent platelet activation^104^. Among these drugs, terutroban and picotamide have been investigated in patients with cerebrovascular disease or PAD (see section below). Pharmacodynamic studies have evaluated EV-077, a thromboxane receptor antagonist and thromboxane synthase inhibitor, in patients with diabetes and stable CAD receiving monotherapy with aspirin or clopidogrel. In this setting, EV-077 provided further platelet inhibition through multiple signalling pathways; however, no specific in vivo data are available for EV-077^105^. Ridogrel, a thromboxane A_2_ (TXA_2_) synthase inhibitor and TXA_2_–prostaglandin endoperoxide receptor antagonist, was investigated in 907 patients with MI treated with thrombolysis and enrolled in the RAPT study^106^. Ridogrel was found to be not superior to aspirin in increasing the fibrinolytic efficacy of streptokinase, as assessed before discharge. In a post hoc analysis, fewer recurrent ischaemic cardiac events occurred in the ridogrel group, without an excess in serious bleeding complications. No specific data in the subgroup with diabetes are available from this investigation.

### Targeting thrombin after ACS

Despite DAPT, patients with diabetes and established atherosclerosis have a high residual risk of recurrent events^87^. This situation is likely to be the direct consequence of endothelial dysfunction, oxidative stress, vascular inflammation, abnormal platelet reactivity, and decreased responsiveness to antiplatelet agents, but increased thrombin generation might also have a role^107^. Thrombin is a potent platelet agonist; therefore, thrombin inhibition might provide substantial suppression of platelet function in addition to reduced fibrin production. Inhibition of the effects of thrombin can be achieved by direct thrombin inhibitors, such as bivalirudin or dabigatran; by direct factor Xa inhibitors, such as apixaban, edoxaban, or rivaroxaban; or by platelet proteinase-activated receptor 1 (PAR1) antagonists, such as vorapaxar.

The effects of thrombin inhibition on cardiovascular outcomes in patients with ACS have been investigated in the ATLAS ACS–TIMI 46 trial^108^ with rivaroxaban (2.5 mg twice daily) and in the TRA 2°P-TIMI 50 trial^109^ with vorapaxar. In both studies, the newer agent under investigation was compared with placebo on a background of DAPT with aspirin plus clopidogrel. In the ATLAS ACS–TIMI 46 trial^108^, no significant interaction was observed between prevention of MACE, reduction in mortality, and increase in bleeding by rivaroxaban and diabetic status. In the TRA 2°P-TIMI 50 trial^109^, the net clinical benefit with vorapaxar in the population with previous MI was numerically more pronounced in patients with diabetes (HR 0.77, *P* = 0.006) than in those without diabetes (HR 0.88, *P* = 0.04). Various factors have precluded the widespread integration of the findings from these two studies into clinical practice. These factors include the delayed initiation of the additional antithrombotic agent after the coronary event, the lack of comparison data between rivaroxaban or vorapaxar in addition to DAPT with aspirin plus clopidogrel or aspirin plus either prasugrel or ticagrelor, and the bleeding concerns associated with triple antithrombotic therapy.

## Secondary prevention after stroke or TIA

Diabetes is independently associated with an increased risk of ischaemic stroke, and the presence of diabetes after a stroke negatively affects recovery and predisposes to recurrent cerebral events^110^. Despite advances in pharmacotherapy, the optimal antiplatelet regimen for patients with diabetes and cerebrovascular disease (with or without concomitant CAD) remains debated because the use of more potent antithrombotic drugs after a stroke or transient ischaemic attack (TIA) might cause a considerable increase in the risk of intracranial bleeding^111^, casting uncertainty on the risk–benefit ratio in the setting of secondary prevention. No specific, large study on aspirin for secondary prevention of stroke in patients with diabetes exists, so the available data come from subgroup analyses of more general studies. Two large meta-analyses of cardiovascular secondary prevention trials, including patients with a previous stroke, indicate that the clinical benefit of aspirin compared with placebo is consistent in patients with or without diabetes^36,64^. As mentioned, in the CAPRIE trial^65^, the reduction of cardiovascular events with clopidogrel versus aspirin was more pronounced in patients with diabetes, and one-third of the patients in CAPRIE had a previous stroke or TIA.

The concept that the residual risk of recurrent events in patients with diabetes might be related to higher on-treatment platelet reactivity when receiving single antiplatelet therapy has led to investigations aimed at assessing whether DAPT or the newer P2Y_12_ inhibitors are associated with an increased clinical benefit in patients with diabetes and cerebral events. In randomized trials, the combination of low-dose aspirin and extended-release dipyridamole (ERDP) (400 mg daily) after a stroke showed a clinical benefit compared with aspirin alone in the ESPS-2 study^112^ and the ESPRIT trial^113^, in which patients with diabetes composed 15% and 18% of the trial cohorts, respectively. Conversely, outcomes after ischaemic stroke were similar with aspirin plus ERDP versus clopidogrel in the PRoFESS trial^114^ regardless of diabetic status (prevalence of diabetes 28%). The use of ERDP in clinical practice is currently discouraged and limited by the high incidence of adverse effects, such as headache and gastrointestinal symptoms.

In the CHARISMA trial^67^, >40% of patients had diabetes and approximately 36% had a previous stroke or TIA at randomization. In the subgroup analysis restricted to 4,320 patients with previous ischaemic stroke or TIA, the addition of clopidogrel was associated with a trend towards a reduction in recurrent stroke (4.9% versus 6.1%; HR 0.80, 95% CI 0.62–1.03)^115^. Among the overall population of the CHARISMA study^67^, the incidence of intracranial bleeding was similar (0.3%) in patients receiving aspirin plus clopidogrel or aspirin alone, and no significant difference in severe bleeding emerged in the subgroup analysis of patients with prior cerebrovascular disease (1.7% with placebo versus 1.9% with clopidogrel; HR 1.11, 95% CI 0.71–1.73)^115^. The efficacy and safety profile of the aspirin plus clopidogrel combination from these analyses formed the basis for subsequent specific randomized studies.

The MATCH trial^116^ investigators randomly allocated patients with stroke or TIA and at least one cardiovascular risk factor to clopidogrel versus aspirin plus clopidogrel. No significant reduction in stroke recurrence was observed in the DAPT group, whereas bleeding complications were increased. These findings were consistent in groups defined according to diabetes status. Conversely, in the CHANCE trial^117^, selectively performed in a Chinese population of 5,170 patients with minor stroke or TIA, DAPT with aspirin plus clopidogrel, given for 21 days after the index event, decreased the rate of recurrent stroke at 90 days compared with aspirin alone, without a significant increase in bleeding events, irrespective of diabetes status. The POINT trial^118^ investigated aspirin alone (50–325 mg daily) versus aspirin plus clopidogrel in patients with minor ischaemic stroke or high-risk TIA. The primary efficacy end point was a composite of ischaemic stroke, MI, or vascular death at 90 days. In the DAPT group, a significant 25% relative reduction in the main outcome measure and a 2.3-fold increased risk of major bleeding were observed. However, no outcome data according to diabetes status are so far available.

Terutroban, a selective thromboxane–prostaglandin receptor antagonist, was compared with aspirin in the PERFORM trial^119^ for the prevention of cerebral and cardiovascular events in patients with previous stroke or TIA. The incidence of the primary end point (a composite of ischaemic stroke, MI, or vascular death, not including haemorrhagic death) at 28 months was 11% in both groups (HR 1.02, 95% CI 0.94–1.12). Similar results were obtained in the subgroup of 5,299 patients with diabetes. However, a significant increase in any bleeding events was observed in patients receiving terutroban (HR 1.09, 95% CI 1.01–1.17); therefore, this agent is not an alternative to aspirin in patients with cerebral ischaemic events.

In the context of the newer, more potent antiplatelet agents, a history of any stroke or TIA is a contraindication for prasugrel therapy. Conversely, ticagrelor, which is contraindicated only after a haemorrhagic stroke, has been tested (180 mg loading dose followed by 90 mg twice daily for 90 days) versus aspirin in 13,199 patients within 24 h of a non-severe stroke or high-risk TIA in the SOCRATES trial^120^. Ticagrelor was not superior to aspirin in reducing the primary end point of stroke, MI, or death at 3 months in either the overall population or the subgroup (25% of patients) with diabetes (8.4% versus 9.5%; *P* = 0.99).

In summary, the available data do not provide clear evidence in support of DAPT (that is, aspirin plus clopidogrel versus aspirin or clopidogrel alone) or of any more potent antiplatelet agent in patients with diabetes and ischaemic cerebral events. Antiplatelet approaches in this setting are shown in Table 3.

## Secondary prevention in PAD

Up to one-third of patients with PAD have diabetes^121^. The prevalence of PAD is higher among patients with diabetes, and in these individuals, PAD is more severe, progresses more rapidly, and entails a higher risk of recurrent ischaemic events and amputation than in those without diabetes^122^. In addition, PAD is a potent marker of systemic atherosclerosis and increased cardiovascular risk^122^. Data on antiplatelet strategies in this setting are limited and are often derived from subgroup analyses of investigations devoted primarily to the management of patients with CAD, in whom PAD was often asymptomatic or not clinically apparent.

In a meta-analysis of 18 trials, involving approximately 5,000 patients with PAD, those receiving aspirin had a modest, nonsignificant 12% lower risk of cardiovascular events than those receiving placebo^123^. This analysis was heavily driven by two studies performed on patients with diabetes, the POPADAD study^47^ (*n* = 1,276) and the VA-Cooperative trial^124^ (*n* = 231), which both showed no benefit of aspirin or of aspirin plus dipyridamole versus placebo. Various aspects might explain the apparent lower efficacy of aspirin in patients with PAD and diabetes. First, current evidence might be underpowered. Alternatively, or additionally, these patients might have phenotypic and biological differences from those with predominant CAD. For instance, the presence of PAD might render platelet activation more critically dependent on ADP than on TXA_2_ production^104^. Third, as stated above, the pharmacodynamics of aspirin might differ in patients with diabetes compared with those without diabetes^24^. Finally, the hypothesis that the inhibition of COX1-dependent TXA_2_ biosynthesis is insufficient in this setting is substantiated by data indicating that the use of picotamide, an inhibitor of both TXA_2_ receptors and TXA_2_ synthase, might increase survival versus aspirin in patients with diabetes and PAD (RR 0.55, 95% CI 0.31–0.98)^125^.

In an analysis of 6,452 patients with PAD included in the CAPRIE trial^65^, approximately one-third of whom had diabetes, clopidogrel recipients had a significant 24% and 22% relative risk reduction in cardiovascular mortality and cardiovascular events compared with aspirin, respectively, with a similar benefit in the subset of patients with diabetes. A post hoc analysis of the CHARISMA study^126^ on 3,096 patients with PAD demonstrated no benefit with aspirin plus clopidogrel versus aspirin alone in terms of reduction of the composite MACE end point; significant decreases in the rates of MI (HR 0.63, 95% CI 0.42–0.96, *P* = 0.029) and hospitalization for ischaemic events (HR 0.81, 95% CI 0.68–0.95, *P* = 0.011) were observed in the DAPT group at the expense of a twofold increased risk of minor bleeding. No specific outcome data are available in the subgroup of patients with PAD and concomitant diabetes in this trial.

The presence of PAD impairs cardiovascular prognosis in patients with a history of MI, leading to the rationale that multi-pathway platelet inhibition might improve clinical outcome in this setting^127^. In the PEGASUS-TIMI 54 trial^128^, 1,143 patients (5%) had known PAD at randomization, and 42% of these had diabetes. Given the high baseline risk profile of this PAD subgroup, the addition of ticagrelor to aspirin (versus aspirin alone) was associated with a greater absolute reduction in MACE in these patients than in patients without PAD (4.1% at 3 years and a number needed to treat of 25 versus 1.0% at 3 years and a number needed to treat of 100) without a significant increase in major bleeding^128^. Of note, the use of ticagrelor in patients with PAD significantly reduced major adverse limb events (a composite of acute limb ischaemia or peripheral revascularization: HR 0.65, 95% CI 0.44–0.95). No specific outcome data with ticagrelor plus aspirin versus aspirin alone have been published in the subgroup of patients with PAD and concomitant diabetes in this trial.

In patients with symptomatic PAD (more than one-third with diabetes) from the EUCLID trial^129^, ticagrelor was shown not to be superior to clopidogrel in reducing major cardiovascular and limb events, with similar rates of major bleeding. Among patients with symptomatic PAD (27% with diabetes) from the TRA 2°P-TIMI 50 trial^130,131^, the use of vorapaxar versus placebo did not significantly decrease MACE but did significantly reduced acute limb ischaemia and the rate of peripheral revascularization; the benefit in limb events was accompanied by an increased risk of bleeding, yielding an uncertain net benefit of vorapaxar in patients with PAD.

While acknowledging the paucity of data from specific studies, indications concerning antiplatelet therapy for cardiovascular prevention in patients with diabetes and PAD reflect the recommendations of the AHA/ACC and ESC guidelines on the general population of patients with PAD^132,133^ (Table 3). The cardiovascular protection afforded by rivaroxaban in addition to aspirin therapy versus aspirin alone in the COMPASS trial^134^ was consistent in the subgroups with PAD (27% of the overall population) or CAD and occurred regardless of diabetic status; in particular, patients with PAD and diabetes receiving rivaroxaban plus aspirin had a twofold higher absolute reduction of the composite end point, including cardiovascular death, MI, and stroke, than patients with PAD but without diabetes. Of note, in the PAD population of the COMPASS study, combination therapy was also associated with a significant 46% relative reduction in major adverse limb events, including major amputation. These findings are likely to influence future guideline recommendations. The ongoing VOYAGER PAD trial^135^, an international, randomized, double-blind, placebo-controlled study, is evaluating the efficacy and safety of the vascular dose of rivaroxaban (2.5 mg twice daily) in patients with symptomatic PAD undergoing peripheral surgical and/or endovascular revascularization^136^.

## Prevention of thromboembolism in AF

Diabetes increases the risk of developing AF and worsens the prognosis of patients with AF^137^. In the ACCORD trial^138^, patients with new-onset AF and diabetes had an increased risk of all-cause death (HR 2.65), MI (HR 2.10), and heart failure (HF) (HR 3.80) compared with those without diabetes. The presence of diabetes raises the incidence of thromboembolic events (stroke or systemic embolism) in patients with AF, explaining the inclusion of diabetes in contemporary scores for stroke prediction^139^. In a meta-analysis of seven studies including >12,000 patients with AF, an overall 70% relative increase in the risk of thromboembolic complications was observed in patients with versus those without diabetes, with a yearly incidence ranging from 3.6% to 8.6%^140^. This variability reflects differences in study designs, definitions of outcome measures, patients’ baseline risk profile, concomitant therapies, and types of diabetic populations included. Analyses have identified a duration of diabetes >3 years as an independent predictor of ischaemic stroke, which is a stronger predictor than glycaemic control^141^. A subgroup analysis from the European PREFER in AF registry, involving >5,000 patients with AF, showed that patients with diabetes receiving insulin therapy had a more than twofold higher risk of stroke or systemic embolism at 1 year than patients without diabetes, whereas diabetes not treated with insulin did not entail a significantly increased risk^142^.

**Fig. 3:**
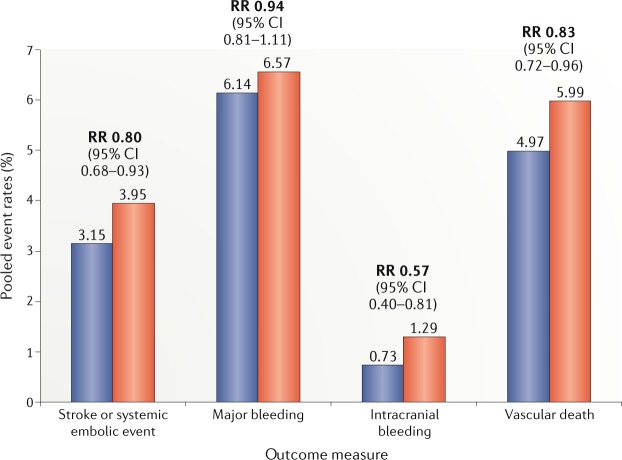
Anticoagulation in patients with diabetes mellitus and atrial fibrillation. Pooled event rates of the various outcome measures from phase III trials comparing non-vitamin K antagonist oral anticoagulants (blue) versus warfarin (red) for the treatment of patients with diabetes and atrial fibrillation. RR, risk ratio.

Oral anticoagulant therapy, historically with warfarin, is the cornerstone of treatment to reduce thromboembolic risk in patients with AF, including those with diabetes^143^. In the past decade, NOACs have revolutionized antithrombotic therapy to prevent AF-related thromboembolic events because of their favourable characteristics: predictable dose–response, rapid offset and onset, fixed doses, no interaction with food, limited interactions with other drugs, and no need for routine monitoring. A meta-analysis of four phase III trials comparing NOACs versus warfarin in patients with AF showed NOACs to be associated with a significant 19% relative reduction in the combined end point including any stroke or systemic embolism (*P* < 0.0001) and a 14% relative reduction in major bleeding (*P* < 0.06) compared with well-managed warfarin^144^. Among 71,683 patients receiving NOACs, the prevalence of diabetes ranged from 23.3% (in the RE-LY study) to 40.0% (in the ROCKET-AF study). Of note, in these trials, blood glucose levels and HbA_1c_ values were not routinely available, and patients with a creatinine clearance <30 ml/min were excluded. Therefore, patients with severe diabetic nephropathy, who are at even higher risk of cardiovascular complications, were excluded^145^. In these phase III studies, no interaction was found between diabetes status and clinical efficacy of the NOACs versus warfarin, although the safety superiority of apixaban versus warfarin was lost among patients with AF and diabetes (*P* = 0.003 for interaction)^145^. Moreover, a study-level meta-analysis of these trials added more robust evidence on the topic^146^ (Fig. 3). Compared with warfarin, the use of NOACs decreased the rates of intracranial bleeding regardless of diabetic status (43% relative reduction versus warfarin in patients with diabetes and 38% in those without diabetes; *P* = 0.47 for interaction)^146^. Also, in patients with diabetes, use of NOACs versus warfarin significantly reduced the rate of cardiovascular death (RR 0.83, 95% CI 0.72–0.96, *P* = 0.01)^146^. To date, no data on a direct comparison between different NOACs in patients with diabetes are available; therefore, the choice of NOAC in patients with diabetes is not supported by specific evidence but should be guided by general principles and take into account the comorbidities associated with diabetes. On the basis of the available data, we recommend antithrombotic strategies for patients with AF and diabetes (Table 3).

## Prevention and treatment of VTE

VTE encompasses a spectrum of diseases that extend from deep-vein thrombosis to pulmonary embolism. Patients with diabetes are characterized by a prothrombotic milieu and have an increased risk of incident and recurrent VTE^147–149^. However, the role of diabetes as an independent predictor of unprovoked or idiopathic VTE has been questioned^150^. Overall, diabetes is now considered only a weak predisposing factor for VTE, and from a practical point of view, its presence does not influence standard antithrombotic treatment^151^. Accordingly, no specific subgroup analyses have been performed on patients with diabetes in VTE trials evaluating the efficacy of vitamin K antagonists or aspirin versus placebo^152^ or, lately, in trials comparing NOACs and warfarin^153^.

**Fig. 4:**
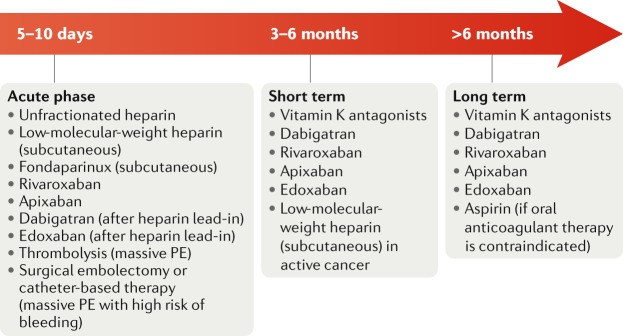
Antithrombotic therapies in patients with diabetes mellitus and venous thromboembolism. Recommendations on antithrombotic strategies for the prevention and treatment of venous thromboembolism in patients with diabetes are similar to those for the general population. PE, pulmonary embolism.

Figure 4 indicates the different types of antithrombotic therapies at different time points after a VTE episode. Evaluation of diabetic status, as well as of other venous and arterial risk factors, is relevant in order to offer appropriate general lifestyle and prevention measures. Indeed, the presence of diabetes might guide the choice of oral anticoagulant therapy, call for closer patient follow-up, or act as a warning for thromboprophylaxis. Given the increased risk of major bleeding in patients with diabetes, NOACs might be the preferred therapeutic option, considering their better manageability and safety profile, especially in an outpatient setting^153^. Moreover, patients with diabetes require closer follow-up because they more frequently develop kidney disease or events related to concomitant PAD or CAD. These conditions might require the introduction of antiplatelet treatment even during the course of anticoagulation for a VTE episode. Patients with diabetes are often receiving polypharmacy; however, no interaction has been reported between the effects of NOACs and glucose-lowering drugs^154^. Finally, a contemporary issue is the real-world underutilization of thromboprophylaxis in vulnerable patient populations in the acute hospital care setting^155^. Owing to the prothrombotic status documented in patients with diabetes, thromboprophylaxis with anticoagulants should be strongly encouraged in these patients, when indicated.

## New glucose-lowering drugs and thrombosis

The evidence reviewed in this Consensus Statement clearly identifies the alterations in mechanisms of thrombosis as important pathological factors for the development of macrovascular complications in patients with diabetes. Nevertheless, the effects of antidiabetic drugs on the pathophysiology of thrombosis are mostly unknown. Previous studies suggested that metformin decreases platelet activation^156,157^ through various mechanisms, including protection of mitochondrial function, reduction of membrane damage, and prevention of oxidative stress^158,159^. Similarly, thiazolidinediones have been shown to reduce prothrombotic mechanisms by modulating platelet function through the activation of 5ʹ-AMP-activated protein kinase and of peroxisome proliferator-activated receptor-γ^160–163^.

In the past decade, various new compounds with different mechanisms of action have been developed to counteract impairments in glucose metabolism^164^. Indeed, since 2008, regulatory agencies have requested pharmaceutical companies to perform randomized, controlled trials with cardiovascular events as outcome measures specifically to characterize the cardiovascular profile of new glucose-lowering drugs. Various studies have been completed (Table 5), and others are ongoing or being planned. Although their direct effects on coagulation and platelet activity are so far unknown, analysis of the available results from the cardiovascular outcome trials might help to identify the agents that are most likely to affect thrombosis in diabetes.

**Table 5:** Cardiovascular outcome trials with the new glucose-lowering drugs

Trial	Drug	Number of patients	Overt CVD (%)	Median follow-up (years)	End point (HR, 95% CI)					Refs
					Primary composite	Cardiovascular death	MI	Stroke	HHF	
*DPP4 inhibitors*										
EXAMINE	Alogliptin	5,380	100	1.5	0.96 (≤1.16)^a^	0.79 (0.60–1.04)	1.08 (0.88–1.33)^b^	0.91 (0.55–1.50)^b^	1.07 (0.79–1.46)	^165^
SAVOR-TIMI 53	Saxagliptin	16,492	79	2.1	1.00 (0.89–1.12)	1.03 (0.87–1.22)	0.95 (0.80–1.12)	1.11 (0.88–1.39)	1.27 (1.07–1.51)^c^	^166^
TECOS	Sitagliptin	14,671	100	3.0	0.98 (0.88–1.09)	1.03 (0.89–1.19)	0.95 (0.81–1.11)	0.97 (0.79–1.19)	1.00 (0.83–1.20)	^167^
*GLP1R agonists*										
ELIXA	Lixisenatide	6,068	100	2.1	1.02 (0.89–1.17)	0.98 (0.78–1.22)	1.03 (0.87–1.22)	1.12 (0.79–1.58)	0.96 (0.75–1.23)	^173^
LEADER	Liraglutide	9,340	81	3.8	0.87 (0.78–0.97)^c^	0.78 (0.66–0.93)^c^	0.86 (0.73–1.00)^c^	0.86 (0.71–1.06)	0.87 (0.73–1.05)	^171^
SUSTAIN	Semaglutide	3,297	83	2.1	0.74 (0.58–0.95)^c^	0.98 (0.65–1.48)	0.74 (0.51–1.08)^b^	0.61 (0.38–0.99)^b,c^	1.11 (0.77–1.61)	^172^
EXCEL	Exenatide	14,752	73	3.2	0.91 (0.83–1.00)	0.88 (0.76–1.02)	0.97 (0.85–1.10)	0.85 (0.70–1.03)	0.94 (0.78–1.13)	^174^
*SGLT2 inhibitors*										
EMPA-REG OUTCOME	Empagliflozin	7,020	>99	3.1	0.86 (0.74–0.99)^c^	0.62 (0.49–0.77)^c^	0.87 (0.70–1.09)	1.18 (0.89–1.56)	0.65 (0.50–0.85)^c^	^169^
CANVAS Program	Canagliflozin	10,142	66	2.4	0.86 (0.75–0.97)^c^	0.87 (0.72–1.06)	0.89 (0.73–1.09)	0.87 (0.69–1.09)	0.67 (0.52–0.87)^c^	^170^

In all trials, the primary end point was a composite of cardiovascular death, nonfatal myocardial infarction (MI), and nonfatal stroke. In the TECOS trial^167^, hospitalization for unstable angina was also included in the composite primary outcome. CVD, cardiovascular disease; DPP4, dipeptidyl peptidase 4; GLP1R, glucagon-like peptide 1 receptor; HHF, hospitalization for heart failure; SGLT2, sodium/glucose cotransporter 2.

^a^Upper boundary of the one-sided repeated confidence interval, at an α level of 0.01.

^b^Hazard ratio for nonfatal events only.

^c^*P* < 0.05 for superiority.

The EXAMINE^165^, SAVOR-TIMI 53 (ref.^166^), and TECOS^167^ studies were among the first cardiovascular outcome trials evaluating a new class of glucose-lowering drugs, the dipeptidyl peptidase 4 (DPP4) inhibitors. All these studies met the primary noninferiority end point for cardiovascular safety of these drugs versus placebo in patients with diabetes and established cardiovascular disease. However, the use of DPP4 inhibitors was not associated with significant benefits on the prespecified measures of cardiovascular outcome. Conversely, a signal of increased risk of HF with saxagliptin was highlighted in the SAVOR-TIMI 53 trial^166^, and a post hoc analysis of the EXAMINE trial^168^ indicated a nonsignificant 0.9% higher absolute incidence of hospitalization for HF in the alogliptin group.

The results of the EMPA-REG OUTCOME trial^169^ were a major breakthrough in the field, showing a significant 14% relative risk reduction in the rate of the composite primary end point, including cardiovascular death, MI, or stroke, with empagliflozin, an inhibitor of the sodium/glucose cotransporter 2 (SGLT2), causing glycosuria. Favourable cardiovascular effects have been subsequently obtained with another drug in the same class, canagliflozin^170^. Of note, the use of either empagliflozin or canagliflozin led to a significant decrease in hospitalizations for HF.

A lower incidence of cardiovascular events has been demonstrated with two different glucagon-like peptide 1 receptor (GLP1R) agonists, liraglutide and semaglutide^171,172^, but not with lixisenatide^173^. Finally, a borderline significant (*P* = 0.06) 0.9% absolute reduction in the primary cardiovascular outcome measure was found with exenatide in the EXCEL study^174^. Of note, unlike SGLT2 inhibitors, treatment with GLP1R agonists resulted in significant protection from individual components of the composite outcome measure (MI and stroke) but was neutral in terms of rates of HF.

**Fig. 5:**
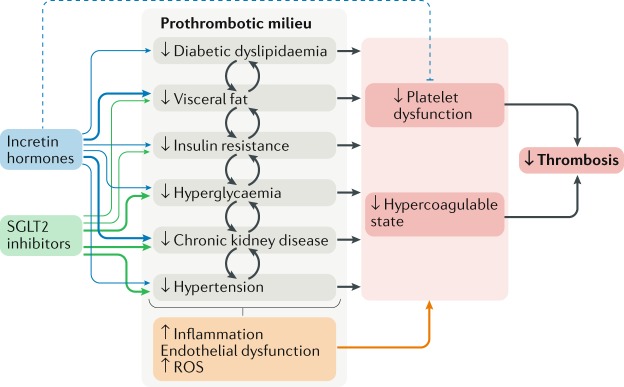
Potential pleiotropic actions of SGLT2 inhibitors and incretin-based therapies for the reduction of thrombotic events. Several conditions contribute to a prothrombotic state in diabetes mellitus, including (but not limited to) hyperglycaemia, hypertension, obesity, insulin resistance, and impaired kidney function. The results of cardiovascular outcome trials and ad hoc studies suggest that both sodium/glucose cotransporter 2 (SGLT2) inhibitors and incretin hormones can counteract these conditions (to different extents and through different pathways), with possible benefits on the diabetic prothrombotic milieu. Some evidence also suggests a direct regulation of platelet activity by glucagon-like peptide 1 (dashed line). Thick lines indicate mainly direct actions; thin lines indicate mainly indirect effects. ROS, reactive oxygen species.

Taken together, these data indicate that, despite a similar control on levels of HbA_1c_, the new glucose-lowering drugs have different effects on cardiovascular outcomes, with potential mechanisms of cardiovascular protection that are independent of glucose control (Fig. 5). The encouraging results of studies evaluating GLP1R agonists suggest that the protection from MI and stroke provided by these drugs derives from effects on the progression and destabilization of atherosclerotic plaques. These results also suggest that the incretin system is involved in the pathophysiology of arterial thrombosis. In this regard, platelets have been shown to express GLP1Rs constitutively, and in vitro stimulation of platelets with liraglutide or exenatide has inhibitory effects on platelet aggregation^175,176^. Conversely, SGLT2 inhibition resulted in a consistent reduction in HF-related events, suggesting that this class of agents acts through different pathways; however, mechanistic studies are needed to clarify the pathways of cardiovascular protection of these compounds. In particular, data on the effect of SGLT2 inhibitors on platelet function and thrombosis are lacking.

## Conclusions

Patients with diabetes have a prothrombotic state and a higher risk of cardiovascular events than those without diabetes. Antithrombotic therapies in these patients might be associated with a higher absolute reduction of recurrent events than in patients without diabetes. More aggressive antithrombotic strategies might be specifically indicated in patients with diabetes and cardiovascular diseases, including the use of the newer, more potent P2Y_12_ inhibitors in patients with ACS, DAPT prolongation beyond 1 year in patients with MI, and short-term DAPT in selected patients with acute ischaemic stroke. However, the expected ischaemic protection from antithrombotic drugs must always be weighed against the risk of drug-related bleeding. Available evidence on the efficacy and safety of antithrombotic drugs in patients with diabetes at risk of or with cardiovascular diseases often derives from underpowered observational studies or subgroup analyses of trials not specifically performed in patients with diabetes; therefore, large investigations specifically focused on patients with diabetes are needed and should be encouraged.
